# Cardiomyocyte-restricted overexpression of extracellular superoxide dismutase increases nitric oxide bioavailability and reduces infarct size after ischemia/reperfusion

**DOI:** 10.1007/s00395-012-0305-1

**Published:** 2012-10-26

**Authors:** Detlef Obal, Shujing Dai, Rachel Keith, Neviana Dimova, Justin Kingery, Yu-Ting Zheng, Jay Zweier, Murugesan Velayutham, Sumanth D. Prabhu, Qianghong Li, Daniel Conklin, Dan Yang, Aruni Bhatnagar, Roberto Bolli, Gregg Rokosh

**Affiliations:** 1 Institute of Molecular Cardiology, Diabetes and Obesity Center, University of Louisville, 580 South Preston Street, Louisville, KY 40202 USA; 2 Department of Anesthesiology and Perioperative Medicine, University of Louisville, Louisville, USA; 3 Department of Physiology and Biophysics, University of Louisville, Louisville, USA; 4 Ohio State University, Columbus, OH USA; 5 University of Hong Kong, Hong Kong, China; 6 Division of Cardiovascular Disease, Department of Medicine, University of Alabama-Birmingham, 311 Tinsley Harrison Tower, 1900 University Blvd, Birmingham, AL 35294-0006 USA; 7 Institute of Molecular Cardiology, Diabetes and Obesity Center, University of Louisville, 570 South Preston Street, Louisville, KY 40202 USA; 8 Institute of Molecular Cardiology, Diabetes and Obesity Center, University of Louisville, 550 South Jackson Street, Louisville, KY 40202 USA; 9 Division of Cardiovascular Disease, Department of Cell, Developmental, and Integrative Biology, University of Alabama-Birmingham, 311 Tinsley Harrison Tower, 1900 University Blvd, Birmingham, AL 35294-0006 USA

**Keywords:** NO-bioavailability, Extracellular superoxide dismutase, Cardioprotection, Ischemia/reperfusion injury, Peroxynitrite

## Abstract

Increased levels of extracellular superoxide dismutase (ecSOD) induced by preconditioning or gene therapy protect the heart from ischemia/reperfusion injury. To elucidate the mechanism responsible for this action, we studied the effects of increased superoxide scavenging on nitric oxide (NO) bioavailability in a cardiac myocyte-specific ecSOD transgenic (Tg) mouse. Results indicated that ecSOD overexpression increased cardiac myocyte-specific ecSOD activity 27.5-fold. Transgenic ecSOD was localized to the sarcolemma and, notably, the cytoplasm of cardiac myocytes. Ischemia/reperfusion injury was attenuated in ecSOD Tg hearts, in which infarct size was decreased and LV functional recovery was improved. Using the ROS spin trap, DMPO, electron paramagnetic resonance (EPR) spectroscopy demonstrated a significant decrease in ROS in Tg hearts during the first 20 min of reperfusion. This decrease in ROS was accompanied by an increase in NO production determined by EPR using the NO spin trap, Fe-MGD. Attenuated ROS in ecSOD Tg myocytes was also supported by decreased production of peroxynitrite (ONOO^−^). Increased NO bioavailability was confirmed by attenuated guanylate cyclase-dependent (p-VASP) signaling. In conclusion, attenuation of ROS levels by cardiac-specific ecSOD overexpression increases NO bioavailability in response to ischemia/reperfusion and protects against reperfusion injury. These findings are the first to demonstrate increased NO bioavailability with attenuation of ROS by direct measurement of these reactive species (EPR, reactive fluorescent dyes) with cardiac-specific ecSOD expression. This is also the first indication that the predominantly extracellular SOD isoform is capable of cytosolic localization that affects myocardial intracellular signal transduction and function.

**Electronic supplementary material:**

The online version of this article (doi:10.1007/s00395-012-0305-1) contains supplementary material, which is available to authorized users.

## Introduction

Extracellular superoxide dismutase (ecSOD) has been found to play an important role in attenuating the effects of the reactive oxygen species (ROS) such as superoxide anion (O_2_^−^), after ischemia/reperfusion injury. Increased oxidative stress caused by ischemia/reperfusion, chronic load, or heart failure leads to myocardial damage and decreased function [6]. EcSOD, which catalyzes the dismutation of O_2_^−^ to H_2_O_2_ and O_2_, is a key enzyme that maintains relatively low levels of this important oxygen-derived radical. Of the three SOD isoforms, ecSOD expression levels are lowest in the heart based on expression levels and relative SOD activity. However, overexpression of ecSOD or treatment with SOD mimetics [31, 32, 60] has been shown to attenuate oxidative stress and to mitigate tissue dysfunction in cardiovascular disease. EcSOD can be upregulated in response to hypoxia and proinflammatory cytokines such as IFN-γ [38]. Angiotensin II induces ecSOD expression through the p42/44 MAP kinase pathway [14], while increased NO production by training upregulates ecSOD through the p38 MAPK pathway [13]. In the vascular wall, vasoactive factors such as histamine, vasopressin, oxytocin, endothelin-1, serotonin and heparin markedly increase enzyme level [57]. Likewise, studies with targeted deletion of ecSOD support the antioxidant role of this enzyme, as the absence of this enzyme was found to exacerbate myocardial dysfunction after myocardial infarction or doxorubicin treatment [29, 59].

Initial hypotheses regarding the mechanism of action of ecSOD focused on attenuation of ROS levels and the consequential oxidation of DNA, lipids, and proteins. Many studies have shown that reperfusion of the ischemic myocardium increases ROS production and that scavenging ROS effectively reverses myocardial injury in animal models [26, 54]. Gene therapy with ecSOD has also been shown to attenuate ischemia/reperfusion injury [31, 32]. While these experimental observations convincingly demonstrate the protective effects of ecSOD, attenuation of ROS has not been successful in moderating dysfunction associated with oxidative stress in clinical scenarios [7].

In addition to the direct actions of ecSOD in attenuating levels of O_2_^−^, indirect effects of attenuated O_2_^−^ have been postulated to lead to an increase in nitric oxide (NO) bioavailability. O_2_^−^ reacts with NO very rapidly, at diffusion-limited rates [15, 17]. The reaction of superoxide with NO leads to the formation of peroxynitrite (ONOO^−^) [30], which is a reactive oxidizing agent. Importantly, early after the identification of endothelial derived relaxation factor (EDRF) as NO, studies in tissues with high ecSOD levels recognized that it is destabilized by O_2_^−^ in vascular bioassays [17, 46]. In these studies, SOD rescued the actions of EDRF/NO generated from endothelial cells, implicating O_2_^−^ in decreased NO bioavailability. In the context of high oxidative stress, the reaction with superoxide exacerbates cellular derangements by removing NO, which would play a beneficial role in several functions in the heart, and by producing ONOO^−^. Further, ONOO^−^ can be protonated to produce O_2_^−^ and nitrogen dioxide, both of which are strong oxidizing species [28, 43]. The action of ecSOD to attenuate O_2_^−^ limits the transformation of NO to ONOO^−^ thereby preserving NO bioavailability for signaling.

NO regulates several systems that affect cardiac function and survival. It is generated by NO synthases or by non-enzymatic reduction of nitrite (for review see [51]). NO is known to play an important role in myocardial protection [24, 50]. NO generated by iNOS and eNOS has been found to be a key mediator of myocardial preconditioning [5]. Recently, we have demonstrated that NO protects the myocardium by limiting ROS formation and thereby preventing mitochondrial permeability transition [62]. NO has been found to modulate contractile function through guanylate cyclase (GC) and PKG [3, 39]. It also exerts direct effects on proteins through the *S*-nitrosothiolation of cysteines [11]. This direct action has been found to modulate the function of several proteins important in myocardial function such as G-protein coupled receptor kinase2 [63], HIF-1α [34], and the ryanodine receptor [64].

To examine the relationship between ROS and NO and to determine whether attenuation of ROS affects NO bioavailability in the heart, we created a cardiac-specific ecSOD transgenic mouse. The effects of increased cardiac-specific ecSOD on ROS and NO were evaluated in vitro and in vivo in the basal state and with oxidative stress. Our findings demonstrate that ecSOD-dependent attenuation of oxidative stress increases NO bioavailability, which translates into improved functional recovery and decreased injury in response to myocardial ischemia/reperfusion.

## Methods and materials

### Materials

Antibodies against phospho-ERK, phospho-AKT, phospho-p38, phospho-JNK, total-ERK, total-AKT, total-p38, total-JNK, phospho-VASP, VASP, phospho-GSK, total GSK, GAPDH, and histone H3 were purchased from Cell Signaling Technology (Beverly, MA, USA). The β-actin antibody was purchased from Sigma Aldrich and the cytochrome C antibody from Abcam. Diethylenetriaminepentaacetic acid was purchased from Sigma Aldrich, USA. The oxygen radical spin trap 5,5-dimethyl-1-pyrroline-*N*-oxide (DMPO) was obtained from Dojindo Laboratories, Kumamoto, Japan. *N*-Methyl-d-glucaminedithiocarbamate (MGD) was synthesized as described previously [53].

### Cardiomyocyte-specific ecSOD transgenic mice

The mouse 5.5 kb αMyHC promoter (kindly provided by Jeff Robbins) [18] was used to direct cardiomyocyte-specific expression of the mouse ecSOD transgene. The mouse ecSOD cDNA was cloned into a SalI–HindIII site of the αMyHC promoter and the linearized construct injected into C57Bl/6 pronuclei. Transgenic offspring were identified by PCR amplification of the transgene. EcSOD Tg mice used in experiments were produced by crossing with WT C57BL/6 mice with littermate WT mice used as controls.

### Langendorff perfused heart preparation for LV function, reperfusion injury, spin trapping and ONOO^−^ measurements

All procedures were conducted under the approval of the University of Louisville IACUC in accordance with the NIH Guide for the Care and Use of Laboratory Animals (DHHS publication No. [NIH] 85-23, rev. 1996) as previously described [9]. LV function, EPR, and reperfusion injury were measured in hearts isolated from WT and ecSOD Tg mice as previously described [62]. The NO spin trap, Fe-MGD (0.5 mM) [67], was administered immediately upon reperfusion (1 ml/min) through a side arm above the aorta cannula. Perfusate was collected at 20-s intervals and immediately frozen in liquid nitrogen. EPR spectra were recorded as described previously [61, 62]. ONOO^−^ release during reoxygenation was measured as luminol chemiluminescence in perfusate collected at reperfusion with infusion of 40 μM luminol (ScienceLab.com, Houston, TX, USA) prepared in 5 mM Na_2_CO_3_, pH 9.2, during the first 2 min of reperfusion as described [61].

### In situ coronary occlusion and infarct size measurement

Hearts were infarcted by coronary artery ligation and reperfused in situ as previously described [9]. Infarct size was assessed 24 h after coronary reperfusion. The non-ischemic region was stained with Evan’s blue (1–1.5 ml, 1 %) injected through the retrobulbar plexus after re-ligation of the coronary artery. The heart was then arrested by intraventricular injection of 0.1 ml KCl (3 M)–CdCl (0.1 M) solution, excised, weighed, rinsed in PBS, and then cut into 1-mm cross-sections. Viable myocardium was determined by TTC staining (1 %, 37° C) for 15 min. Images were digitally acquired and the infarct size determined as previously described [20].

### Cardiac myocyte isolation for hypoxia/reoxygenation studies

Adult cardiomyocytes from WT and ecSOD Tg mice were isolated as previously described [25]. NO bioavailability after hypoxia/reoxygenation was measured in myocytes plated on polycarbonate microwell plates (Nunc, Thermo Fisher) incubated with either the NO sensitive fluorescent dye 4-amino-5-methylamino-2′,7′-difluorofluorescein (DAF-FM, 10 μM, Molecular Probes) for 1 h. ROS and ONOO^−^ were measured using the ROS sensitive dye 5-(and-6)-carboxy-2′,7′-dichlorodihydrofluorescein diacetate (carboxy-DCFDA, 10 μM, Molecular Probes) and ONOO^−^ indicators luminol and HKGreen3 [44] (5 μM, a kind gift from Dr. Dan Yang, University of Hong Kong) for 1 h. The culture medium was then immediately changed to ischemic buffer. Myocytes were treated under hypoxic conditions for 30 and 90 min and reoxygenated as previously described [25]. The microwell plate inserts were placed in normoxic-modified KH buffer containing 1.26 mM Ca^2+^ and the fluorescent signal measured immediately (DCFDA, 495/529 nm abs/em; DAF-FM, 495/515 nm abs/em; HKGreen3, 485/540 nm abs/em; BioTec Synergy 2, Winooski, USA). The signal from each well was subsequently normalized to protein content determined by Bradford assay.

### Western analysis for cell signal transduction

Hearts from WT and ecSOD Tg mice were isolated, frozen, pulverized, and homogenized in buffer and homogenates prepared and processed for PAGE and Western analysis as described previously [25].

### EcSOD activity measurement in whole hearts

Hearts from WT and ecSOD Tg mice were homogenized and sonicated in ice-cold homogenization buffer containing (in mM) 20 HEPES, 1 EGTA, 210 mannitol, and 70 sucrose. SOD activity was determined in a 1,500 g supernatant (Cayman). EcSOD was isolated by concanavalin A-Sepharose 4B (Sigma Aldrich) chromatography [35]. EcSOD was eluted with 0.5 M α-methyl-d-mannoside and SOD activity determined.

### Detection of intracellular ecSOD distribution by immunofluorescence microscopy

Extracellular superoxide dismutase localization in myocytes was assessed by immunofluorescent confocal microscopy (Zeiss LSM 510) in 4 μm paraffin sections stained with anti-rabbit ecSOD-IgG (1:50) and counterstained with FITC-conjugated wheat germ agglutinin (WGA-FITC, Molecular Probes) to detect plasma membrane, and 4′,6-diamidino-2-phenylindole (DAPI) for nuclear staining.

### Statistical analysis

All data are presented as mean ± SEM. Differences between groups were compared by unpaired Student’s *t* test. Comparisons among multiple groups or between two groups at multiple time-points were performed by either one-way or two-way ANOVA followed by paired or unpaired Student’s *t* test with Bonferroni correction. Multiple time-point comparisons between ecSOD Tg and WT mice were performed with two-way repeated-measures ANOVA (Graphpad 5.03, San Diego, CA, USA). A value of *P* < 0.05 was considered significant.

## Results

**Fig. 1:**
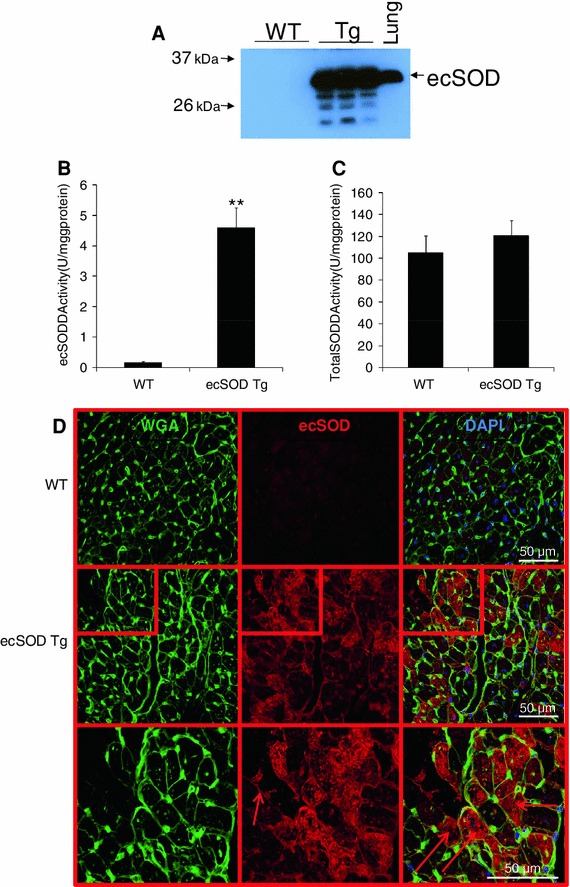
Cardiac myocyte-specific ecSOD overexpression. Mouse ecSOD was overexpressed in cardiac myocytes under the direction of the mouse αMyHC promoter. **a** Western analysis demonstrates a marked increase in ecSOD in ecSOD Tg hearts relative to those of WT littermates. **b** EcSOD was purified from Cu–Zn SOD and Mn-SOD by concanavalin A chromatography. EcSOD activity was significantly increased in transgenic hearts/myocytes. **c** Total SOD activity was slightly, but not significantly, increased in ecSOD Tg hearts. **d** Confocal microscopy localizes transgenic ecSOD to the myocyte cytosolic space (*arrows right magnification*) in addition to the extracellular space (*arrow middle magnification*) defined by wheat germ agglutinin (WGA) staining. Values are the mean ± SEM (*n* = 3). **P < 0.01

Extracellular superoxide dismutase gene therapy and ischemic preconditioning with increased ecSOD have been shown to protect the myocardium from ischemia/reperfusion injury. To further investigate the mechanisms by which ecSOD protects the myocardium from ischemia/reperfusion injury, we generated cardiac-specific ecSOD Tg mice under the direction of the αMyHC promoter. EcSOD expression was increased in heart and cardiac myocytes of ecSOD Tg mice (Fig. 1a; Supplemental Figure 1A) resulting in a 27.5-fold increase in ecSOD activity (Fig. 1b), which, however, did not significantly increase total myocardial SOD activity (Fig. 1c). EcSOD is typically associated with the extracellular matrix due to the presence of a carboxyterminal heparin-binding domain [27, 35]. Low levels of endogenous ecSOD relative to CuZn- or Mn-SOD have led to the conclusion that this isoform is predominantly extracellular [36, 37, 47]. Few studies have demonstrated that ecSOD can also be localized to other subcellular locations [41, 42]. We found that ecSOD was strongly co-localized to the plasma membrane (with WGA) in sections from Tg hearts (Fig. 1d). However, we were surprised to also find ecSOD within the cytoplasm of myocytes in Tg hearts (Fig. 1d). These results suggest that, when overexpressed, ecSOD is not exclusively associated with the extracellular membrane in the heart and may be distributed throughout the cytoplasm.

**Fig. 2:**
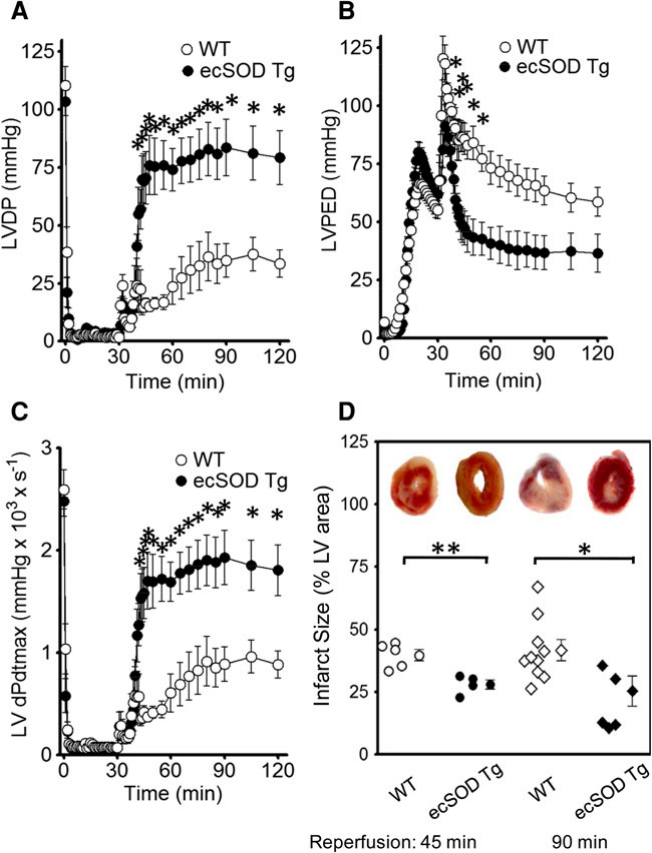
EcSOD overexpression protects the heart from dysfunction and injury following ischemia/reperfusion in vitro. Cardiac function and infarct size were measured in isolated WT and ecSOD Tg hearts perfused using the Langendorff model. Cardiac function **a** LV developed pressure (LVDP), **b** LV end diastolic pressure (LVEDP) and **c** LV d*P/*d*t*_max_ was measured in hearts that had undergone 30 min of global ischemia followed by 90 min of reperfusion at 37 °C. **d** EcSOD overexpression limited infarct size after 30 min ischemia. Infarct size, defined by TTC staining and measured as percent of risk region, was significantly decreased in hearts reperfused for either 45 or 90 min. Values are the mean ± SEM (*n* = 6–11). *P < 0.05, **P < 0.01

**Fig. 3:**
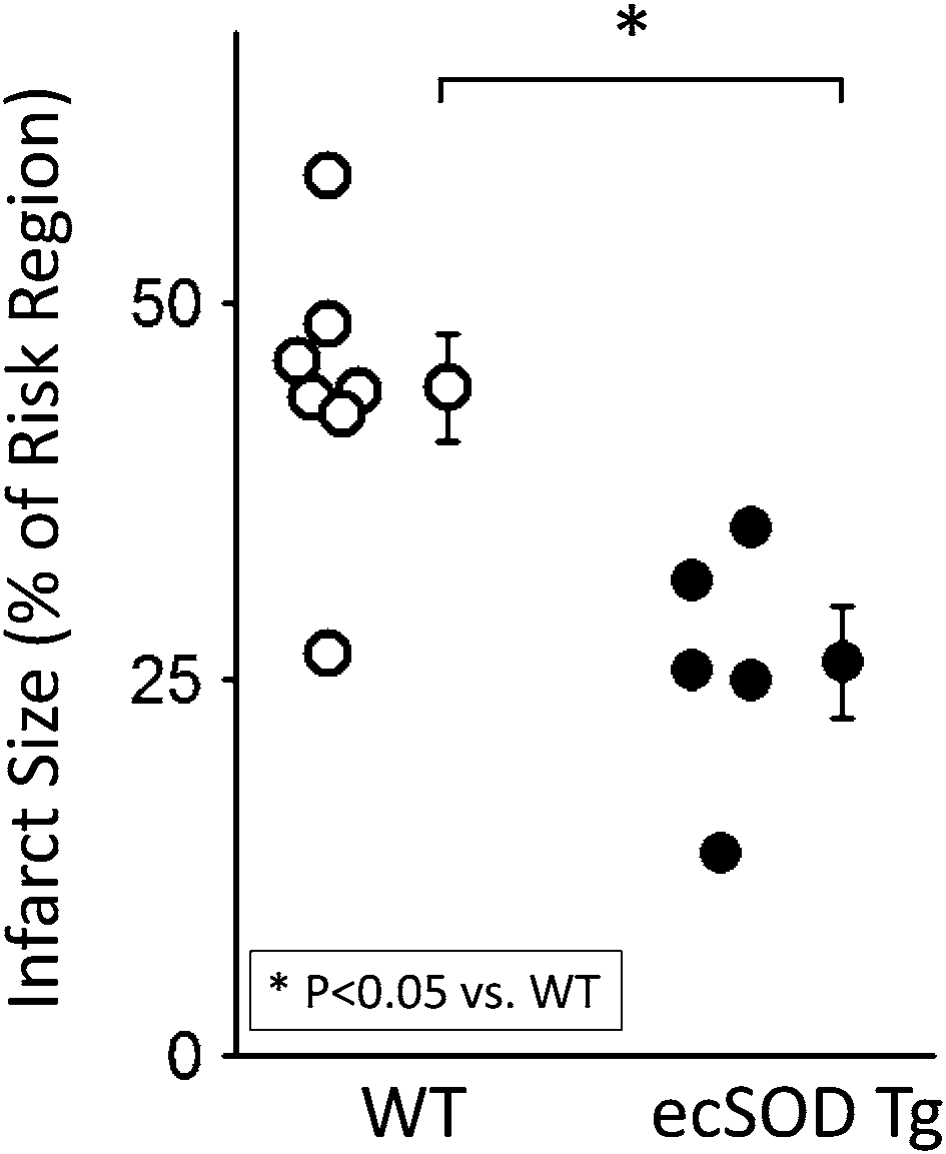
In vivo myocardial ischemia/reperfusion injury is attenuated in the ecSOD Tg mice. In WT and ecSOD Tg mice, a coronary artery was occluded for 30 min and reperfused for 24 h. Risk and infarct areas were determined and infarct size expressed as a percent of risk region. Values are the mean ± SEM (*n* = 6–7). *P < 0.05

To determine whether increased myocyte-specific ecSOD could attenuate ischemia/reperfusion injury, Langendorff perfused WT and ecSOD Tg hearts were subject to 30 min global ischemia followed by 45 or 90 min reperfusion. LV functional recovery after 30 min of global ischemia was significantly improved in ecSOD Tg mice compared to WT (Fig. 2a–c). This improvement in recovery was manifest as increased LV-developed pressure (Fig. 2a), decreased diastolic pressure (Fig. 2b), and increased LV d*P*/d*t*_max_ (Fig. 2c). Infarct size, assessed by TTC staining in WT hearts with 45- and 90-min reperfusion, was similar (Fig. 2d). In ecSOD Tg hearts, infarct size was significantly decreased in both 45- and 90-min reperfusion groups (Fig. 2d). In vivo, coronary artery occlusion resulted in significantly smaller infarcts in ecSOD Tg mice in comparison with WT mice (ecSOD Tg, 26 ± 2 % of the risk region; WT, 45 ± 4 %, *P* < 0.05; Table 1; Fig. 3). Collectively, these data demonstrate that myocyte-specific ecSOD overexpression confers myocardial protection against ischemia/reperfusion injury in vitro and in vivo, resulting in improved functional recovery and decreased infarct size.

**Table 1:** Age, heart sizes, left ventricle size, risk region, infarct area, and temperature and heart rate during ischemia

Phenotype	Age (weeks)	HW/BW	Planimetry results						Heart rate and temperature during ischemia			
			LV	Risk region		Infarct size			15 min		30 min	
			mg	mg	% of LV	mg	% of RR	% of LV	Temp (°C)	HR (bpm)	Temp (°C)	HR (bpm)
WT (*n* = 7)	15 ± 1	4.2 ± 0.1	61 ± 5	28 ± 2	47 ± 5	13 ± 2	45 ± 4	21 ± 3	37.0 ± 0.2	477 ± 15	37.2 ± 0.1	460 ± 31
ecSOD TG (*n* = 5)	22 ± 9	4.9 ± 0.6*	76 ± 3	26 ± 1	38 ± 4	7 ± 0*	26 ± 2*	10 ± 2*	36.9 ± 0.3	474 ± 22	37.1 ± 0.0	494 ± 40

Data are means ± SEM

*BW* body weight, *HW* heart weight, *RR* risk region, *LV* left ventricle, *Temp* temperature taken within the thoracic cavity during coronary occlusion, *bpm* beats/min

* *P* < 0.05 versus WT

**Fig. 4:**
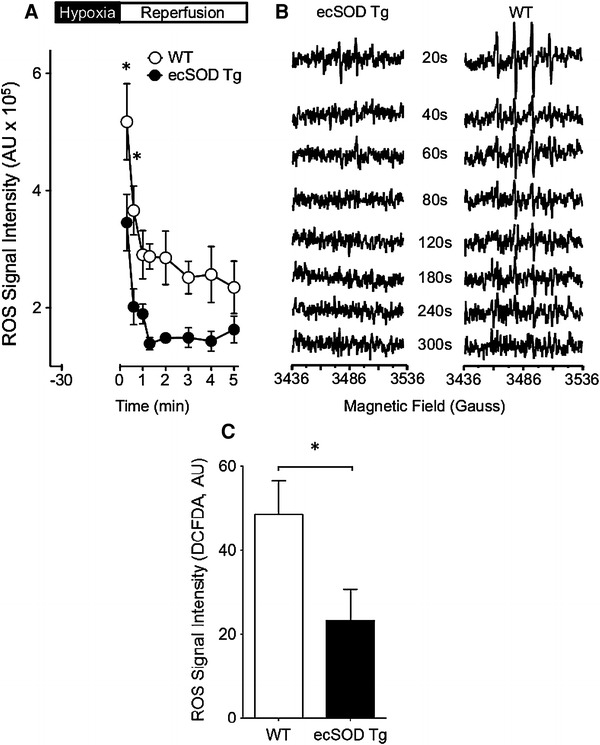
Cardiac-specific ecSOD overexpression attenuates ROS in the heart. **a** ROS were measured by EPR in the perfusates of Langendorff-perfused hearts. Hearts were perfused with the ROS spin trap DMPO immediately upon reperfusion and perfusate was collected for EPR analysis. **b** EPR spectra. **c** The effect of ecSOD overexpression on ROS was verified in isolated cardiac myocytes using the fluorescent ROS indicator DCFDA. Values are the mean ± SEM (*n* = 5–7). *P < 0.05

An increase in ecSOD expression would be expected to diminish ROS levels. To verify this, ROS were measured by EPR spectroscopy in Langendorff perfused hearts. WT hearts perfused with the spin trap, DMPO, at the onset of reperfusion showed a rapid burst of ROS that declined quickly by 80 s (Fig. 4a). The spike in ROS upon reperfusion was significantly attenuated in ecSOD Tg hearts. ROS levels rapidly returned to a stable value in ecSOD Tg hearts and in WT hearts; however, in the ecSOD Tg hearts ROS declined to a lower level than that in WT hearts. To verify that the effect of ecSOD on ROS was specifically associated with myocytes, ROS levels were measured in isolated myocytes using the fluorescent indicator DCFDA under ambient O_2_ levels and after 30 min hypoxia (1 % O_2_). Similar to the results obtained through EPR analysis, ROS levels upon reoxygenation after hypoxia were markedly attenuated in myocytes from ecSOD Tg hearts compared with those from WT hearts (Fig. 4c).

**Fig. 5:**
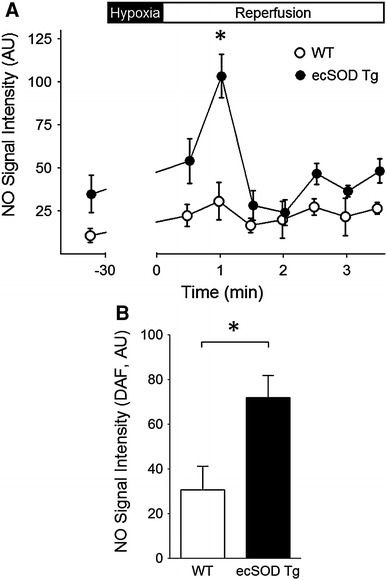
NO bioavailability is increased in ecSOD Tg hearts. **a** NO levels were measured in ecSOD Tg and WT hearts by EPR spectroscopy. Isolated hearts were perfused with the NO spin trap, Fe-MGD, immediately upon reperfusion and the perfusate collected and analyzed by EPR spectroscopy. **b** The effect of ecSOD overexpression on NO bioavailability was verified in isolated myocytes using the fluorescent NO indicator DAFDA. Values are the mean ± SEM (*n* = 6). *P < 0.05

Previous work, primarily in vascular biology, has suggested that a decrease in ROS would lead to an increase in NO bioavailability as a consequence of decreased reaction between O_2_^−^ and NO and decreased formation of ONOO^−^ [17, 46]. This notion of increased NO bioavailability was extrapolated from functional endpoints, such as vessel relaxation. Since ROS levels were attenuated in isolated hearts (by EPR) and in isolated myocytes (by DCFDA fluorescence), we directly measured NO in isolated hearts using the NO spin trap, Fe-MGD, to determine whether the decrease in ROS would be accompanied by an increase in NO. Hearts were perfused with Fe-MGD under normoxic conditions prior to ischemia and at the onset of reperfusion. Baseline NO levels tended to be higher in ecSOD Tg than in WT hearts (*P* = 0.06, Fig. 5a). In ecSOD Tg hearts, NO levels increased dramatically after reperfusion peaking at 60 s and then returning to a level that was slightly elevated compared to WT. In WT hearts, NO levels after reperfusion increased slightly but insignificantly over normoxic baseline levels unlike the peak observed in ecSOD Tg hearts. Protein levels of iNOS and eNOS in ecSOD Tg hearts measured by Western analysis were not different from WT hearts (Supplemental Data, figure 1B).

**Fig. 6:**
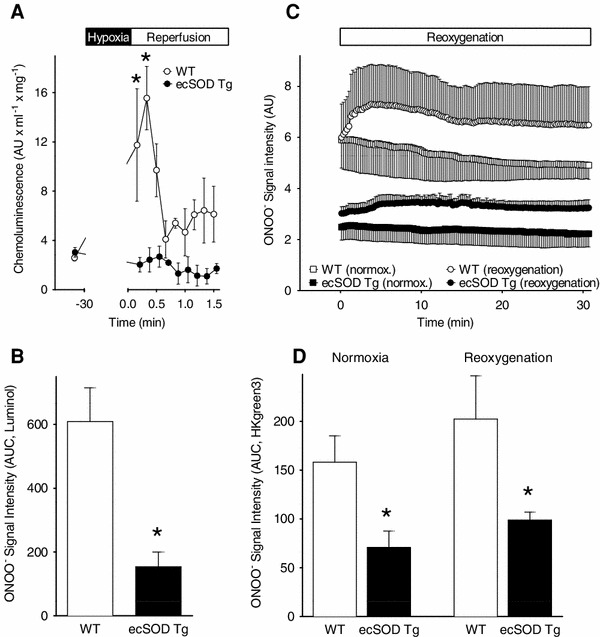
Baseline and hypoxia/reoxygenation ONOO^−^ levels are attenuated in myocytes from ecSOD Tg hearts. ONOO^−^ levels were measured in myocytes isolated from WT and ecSOD hearts detecting luminol chemiluminescence (**a**, **b**) and HKGreen3 fluorescence (**c**, **d**). Values are the mean ± SEM (*n* = 3–4). *P < 0.05

To verify that increased NO was myocyte-specific, NO was measured in myocytes isolated from ecSOD Tg and WT hearts using the fluorescent NO indicator DAFDA. Myocytes constitutively express eNOS and nNOS, associated with caveolae and sarcoplasmic reticulum, respectively, and are capable of producing NO [3, 8]. Upon reoxygenation, NO levels in ecSOD Tg myocytes were significantly higher with reoxygenation than in WT myocytes (Fig. 5b). The marked difference in response to reoxygenation between WT and ecSOD Tg myocytes demonstrates that the attenuation of ROS is associated with increased NO bioavailability and supports the finding of increased NO bioavailability observed in Langendorff perfused hearts. Attenuated ROS levels in the ecSOD Tg heart would lead to decreased levels of ONOO^−^ and decreased injury with reperfusion [61]. ONOO^−^ levels were measured by luminol chemiluminescence [61] and HKGreen3 fluorescence [44] in isolated myocytes from Tg and WT hearts under basal conditions and with hypoxia reoxygenation. ONOO^−^ levels in Tg myocytes were significantly lower than in WT myocytes with luminol and HKGreen3 detection (Fig. 6a, b). The ONOO^−^ burst observed in the first 30 min of reoxygenation was attenuated in myocytes from ecSOD Tg hearts (Fig. 6c, d) with an unchanged, but significantly lower ONOO^−^ release in myocytes of ecSOD transgenic mice during the remaining 90 min of reoxygenation (data not shown). The decrease in ONOO^−^ in myocytes from ecSOD Tg hearts further supports attenuated ROS release with increased ecSOD and provides an additional mechanism for improved protection from reperfusion injury.

**Fig. 7:**
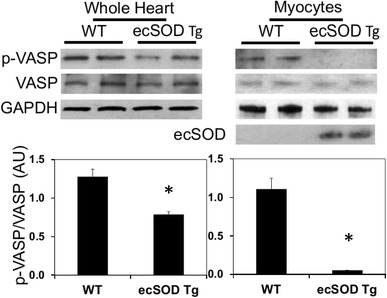
Increased NO bioavailability attenuates GC-dependent signaling. Western blots showing the levels of p-VASP and total VASP in WT and ecSOD hearts. VASP phosphorylation status was used as a measure of GC-dependent signaling. p-VASP and VASP were measured in whole heart (*left panels*) and myocyte (*right panels*) homogenates from WT and ecSOD Tg mice by Western analysis. Values are the mean ± SEM (*n* = 2–4). *P < 0.05

To elucidate the mechanisms by which increased NO and decreased ROS may mediate protection against ischemia/reperfusion injury, we examined NO signaling via a GC-dependent pathway by measuring Ser239 phosphorylation and activation of the cGMP-dependent protein kinase (PKG) substrate vasodilator-stimulated protein (VASP) [55, 56]. VASP levels were similar in hearts from ecSOD Tg and WT mice (Fig. 7). Phosphorylation of VASP ser239, which is the preferential PKG site, was attenuated in ecSOD Tg hearts compared with WT (Fig. 7, left panels) and this was corroborated in isolated myocytes (Fig. 7, right panels). This finding supports the increase in NO bioavailability in ecSOD TG hearts leading to activation and the subsequent desensitization of GC-dependent signaling.

**Fig. 8:**
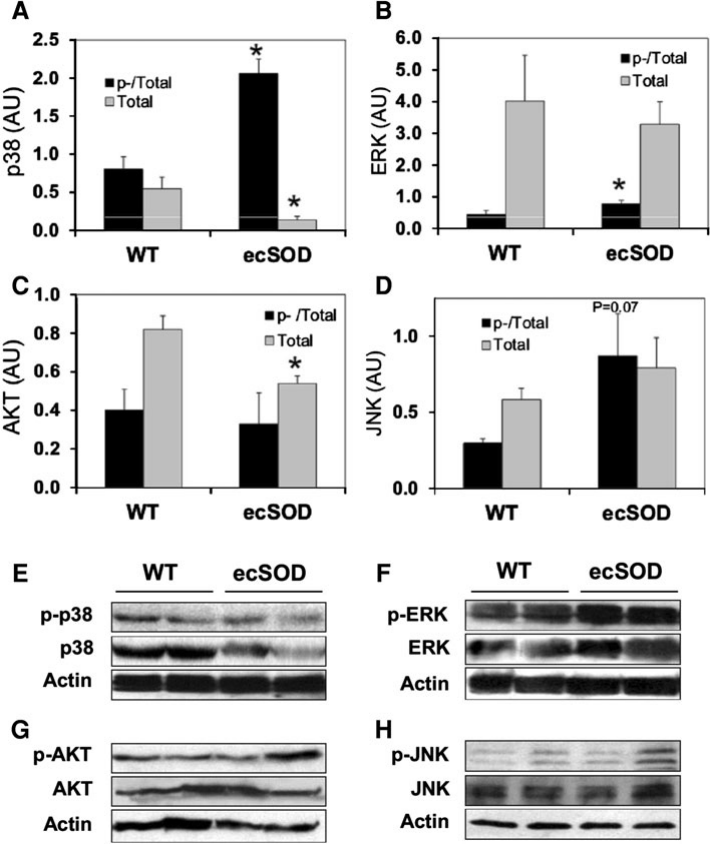
MAPK and AKT signaling in ecSOD Tg hearts. MAPK and AKT phosphorylation were measured in hearts of WT and ecSOD mice by Western analysis. Phospho- and total levels of p38 (**a**), ERK (**b**), AKT (**c**), and JNK (**d**) were measured in hearts of WT and ecSOD Tg mice. Representative Western blots for summary data; p38 (**e**), ERK (**f**), AKT (**g**), and JNK (**h**). Total kinase levels were normalized to β-actin and phospho-kinase levels were normalized to total kinase. Values are the mean ± SEM (*n* = 5–7). *P < 0.05

To investigate further changes in signaling that may contribute to the protective effects of decreased oxidative stress, the status of stress activated and survival signaling through MAPKs and AKT were examined in ecSOD Tg and WT hearts. In whole heart homogenates, baseline levels of total ERK and JNK were unchanged (Fig. 8b, d), whereas, in comparison with WT hearts, p38 and AKT levels were significantly decreased in ecSOD Tg (Fig. 8a, c). Levels of activated phosphorylated p38 and ERK were higher in ecSOD Tg compared to WT hearts (Fig. 8a, b). Levels of p-JNK were elevated nearing significance (*p* = 0.07) (Fig. 8d). Although p-p38 has increased relative to total p38, the overall level of p38 has decreased due to the decrease in total p38 in ecSOD Tg hearts. In response to ischemic stress, in vitro ischemia/reperfusion injury did not significantly affect levels of phosphor-ERK or AKT although total ERK and AKT were slightly increased (Supplemental figure 3).

## Discussion

In this study, we present novel insights into mechanisms that account for ecSOD-dependent myocardial protection against ischemia/reperfusion injury helping to close the gap in understanding the molecular mechanism of ischemia/reperfusion injury [22, 52]. Using a cardiac-specific ecSOD Tg mouse, we demonstrate that ecSOD overexpression leads to both decreased ROS and ONOO^−^ levels and increased NO bioavailability. This increase in NO augmented signaling through a GC-dependent pathway, pVASP, demonstrated by its desensitization, while increasing ERK and decreasing p38 signaling. Our analysis of the ecSOD Tg mouse also demonstrates that increased ecSOD expression results in measurable localization of the protein to intracellular sites. These results constitute the first report of an assessment of the effects of decreased ROS levels on NO levels in the heart, measured directly using spin trapping and a fluorescent indicator. Our data establish a critical relationship between O_2_^−^ scavenging and NO in myocardial tissue, and the importance of this relationship for myocyte signaling and survival after acute ischemia/reperfusion.

We had previously shown that ecSOD gene therapy in the rabbit affords protection against ischemia/reperfusion injury to an extent similar to the late phase of preconditioning [31, 32]. It has also been shown that ecSOD is upregulated by ischemic preconditioning in the heart [40]. To further determine the capacity of ecSOD to protect the myocardium from ischemia/reperfusion injury, we created a cardiac myocyte-specific ecSOD Tg mouse. Our results demonstrate that myocardial ecSOD overexpression attenuates ROS levels in the reperfused heart while affording protection against tissue injury and dysfunction in the post-ischemic heart. These results are significant for several reasons. First, they provide direct evidence that ROS (measured by the O_2_^−^/OH^−^ spin trap DMPO in whole hearts and by the fluorescent ROS indicator DCFDA in isolated myocytes) are decreased by ecSOD. In addition, they suggest that ecSOD may not only exert its effects via its archetypal extracellular localization but may also be present within the cytoplasm exerting influence on O_2_^−^ levels within the myocyte. Myocardial ecSOD expression accounts for the lowest level of SOD activity of the three SOD isoforms and is difficult to detect in some cells/tissues. EcSOD gene therapy in rabbits to limit myocardial injury after infarction showed ecSOD was not expressed in the heart [31], yet was significantly increased due to that secreted by the liver to bind myocardial extracellular matrix [32]. Here total SOD levels were significantly elevated over basal after release with heparin and protamine (~15 U/mg protein). In humans, preconditioning increased significantly ecSOD activity in plasma (~25 U/mg protein) [40]. Unfortunately, myocardial ecSOD could not be measured in this context. Our study demonstrates that very small increases in myocardial ecSOD (~4 U/mg protein) that is distributed through the cytoplasm in addition to the extracellular matrix is capable of conferring protection against ischemic injury. In this context, ecSOD localization at the cell surface due to its carboxyterminal heparin-binding domain [1] may not be the only indicator of ecSOD functionality. EcSOD translocation to the nucleus with oxidative stress [41] and cytoplasmic and vesicular localization in neurons [42] suggest that intracellular effects may be an important functional domain of ecSOD-mediated cardioprotection.

While NO-dependent actions have been studied extensively, the preservation or modulation of the NO signal, its bioavailability, has been relatively less explored due to the challenge of measuring this labile species. The cardiac-specific ecSOD Tg mouse offers an opportunity to determine the effects of modulating O_2_^−^ on NO bioavailability. Direct measurements of NO production by EPR with the spin trap, Fe-MGD, showed that NO bioavailability was increased in ecSOD Tg hearts under basal conditions and NO levels increased dramatically and peaked within 60 s of reperfusion. The rapid rise of NO in the ecSOD Tg hearts is likely a consequence of attenuated ROS levels unmasking NO generation and preservation as a result of decreased inactivation of NO by O_2_^−^ [10]. The increase in NO immediately after reperfusion would be expected to be highly beneficial and contribute to myocardial protection by limiting ROS formation and MPT as we described previously in the cardiac-specific iNOS Tg mouse [62] and with eNOS and iNOS gene therapy [33, 58]. In this scenario, ecSOD as well as increased NO could contribute to attenuating ROS. These findings are notable because levels of NOS isoforms were unchanged in the ecSOD Tg hearts (supplement figure, 1 panels b and c), emphasizing the relationship between ROS and NO. Localization of NOS isoforms, two of which are sensitive to Ca^2+^, (eNOS and nNOS), to myocyte mitochondria has been demonstrated by different groups [16, 21, 66]. Close proximity of ecSOD to these mitochondrial NOS isoforms is therefore, likely to be a key factor in the preservation of NO levels in the reperfused heart. This view is supported by our findings that ecSOD is also localized to the cytoplasm as well as membrane domains, where the greatest levels of ROS would be expected to be generated during reperfusion. It is also important to note that increased NO bioavailability should be cardiac-restricted as increased NOS in blood cells may exacerbate injury [19].

Increased NO bioavailability and increased ability of NO to impact its downstream signaling pathway were verified by examining GC-dependent signal transduction in hearts from ecSOD Tg mice. The decrease in levels of GC-dependent VASP phosphorylation demonstrated the functional consequences of increased NO bioavailability. The decrease in VASP phosphorylation and activation is a consequence of soluble GC desensitization with increased NO levels [4, 65]. This observation provides functional support for the preservation of NO via decreased ROS and provides a new paradigm in which superoxide levels regulate not only the extent but also the nature of NO signaling. In the context of high levels of SOD activity from both Cu,Zn- and Mn-SOD, this finding may suggest that ecSOD may have different intracellular targets and thus exert its effects within different intracellular domains. *S*-nitrosocysteine levels in whole hearts and isolated myocytes were examined by the biotin switch method, however, results did not demonstrate an increase in total levels (data not shown). This does not, however, preclude increased *S*-nitrosylation of specific proteins which may confer cardioprotection through increased NO bioavailability. These observations fit the overall paradigm of increased NO bioavailability occurring as a result of increased ecSOD, and suggest that cardioprotection occurs as a consequence of GC-dependent signal transduction.

Concomitant with NO-dependent signaling, decreased oxidative stress impacted MAPK and AKT signaling, which are typically associated with cell stress and survival. ROS have been shown to activate MAPKs in cardiac myocytes and fibroblasts and increases in activated p-ERK, p-AKT [12] and p-p38 [48, 49] are characteristically known to promote cell survival [2]. In this respect, the protection from ischemia/reperfusion injury is expected. However, increased activation of these signaling pathways would be contrary to expectations if it were based exclusively on ROS levels. Considering studies in ecSOD KO mice, the decrease in p-p38 in the ecSOD Tg is the only observation congruent with increased activation of p38 in the KO mice. The complexity of p38 changes in the ecSOD Tg heart, increased p-p38 in relation to total p38, may suggest preferential activation of p38β in the context of increased protection [49]. Increased ERK and JNK activation are similar to that seen in the ecSOD KO [59]. This suggests ERK and AKT may be activated by a mechanism independent of increased ROS, as after ischemia/reperfusion both ERK and AKT phosphorylation were unchanged (supplement figure 3, panels A–C). NO has been shown to have a direct effect on PKC increasing its translocation and activation via tyrosine nitration [16]. This activation results in complex formation and activation of AKT [66] as well as ERK and JNK [37]. These studies provide a conceptual basis for our findings that increased NO bioavailability with decreased oxidative stress in the ecSOD Tg leads to activation of these stress-related kinases. P38 has been shown to be ubiquitinated and degraded by the proteasome in a c-Jun-dependent manner [45]; however, no direct evidence exists to support a role for NO or redox balance. Overall, these changes in ERK, AKT, JNK and p38 would support a pro-survival environment and would be expected to contribute to cardioprotection in the ecSOD Tg.

In conclusion, the main novel findings of this study can be summarized as follows. The cardiac-specific ecSOD transgenic mouse described in this study provides useful insights into the relationship between oxidative stress and NO bioavailability. Using luminol chemiluminescence, the spin traps, DMPO and Fe-MGD, and the reactive fluorescent dyes, DCFDA, DAF and HKGreen3, to directly measure ROS, NO and ONOO^−^, we show for the first time that cardiac myocyte specific ecSOD overexpression attenuates ROS and ONOO^−^ and that this results in a concomitant increase in NO. EcSOD, previously considered to be exclusively extracellular, is capable of localizing to intracellular domains and although transgenic ecSOD constitutes a small fraction of total myocardial SOD activity, it is capable of attenuating overall oxidant stress in the heart, increasing NO bioavailability resulting in protection against ischemia/reperfusion injury both in vitro and in vivo. The augmentation of NO by ecSOD is sufficient to impact NO-dependent GC-signaling pathways. Finally, the results demonstrate that although ecSOD is considerably less abundant than other SOD isoforms in the heart, this enzyme plays a critically important role as an arbiter of cardiac antioxidant defenses and NO-dependent actions, and that selective upregulation of ecSOD is capable of significantly reducing myocardial oxidative stress and alleviating ischemia/reperfusion injury. The observation of intracellular localization of ecSOD provides a potential novel mechanism of action that needs to be addressed in future studies.

## Electronic supplementary material

Below is the link to the electronic supplementary material.

**Supplement Figure** 1. EcSOD protein expression in isolated cardiomyocytes and whole heart from WT and ecSOD Tg mice detected by Western analysis (panel A); Representative Western-blots and quantitative analysis of eNOS expression in whole heart from WT and ecSOD Tg mice detected by Western analysis (n = 3, panel B). iNOS expression in whole heart from WT and ecSOD Tg mice. Protein samples from fresh isolated lung and heart tissue of cardiomyocyte-specific iNOS overexpressing mice [23] served as positive control. GAPDH was used as loading control (panel C)**Supplement Figure** 2. Confocal microscopy images of isolated cardiomyocytes of WT (A) and ecSOD Tg mice (B) after 30 min of hypoxia and 15 min of reoxygenation. Experiments were performed after 1 h incubation in HKGreen-3 (5 μM), Mitosox Red (5 μM) and Hoechst stain (5 μM)**Supplement Figure** 3. ERK and AKT signaling in isolated Langendorff-mode perfused WT and ecSOD Tg hearts. Phospho- and total levels of AKT (panel A) ERK (panel B) were measured in hearts of WT and ecSOD Tg mice after 30 min global ischemia and 90 min reperfusion. Representative Western blots for summary data (panel C). Total kinase levels were normalized to GAPDH and phospho-kinase levels were normalized to total kinase. Values are the mean ± SEM (n = 3). *P < 0.05 vs. WT

## Abbreviations

αMyHC: Alpha-myosin heavy chain
DAF-FM: 4-Amino-5-methylamino-2′,7′-difluorofluorescein (fluorescent NO probe)
DCFDA: 5-(and-6)-Carboxy-2′,7′-dichlorodihydrofluorescein diacetate (fluorescent ROS probe)
DMPO: 5,5-Dimethyl-1-pyrroline-*N*-oxide (ROS spin trap)
ecSOD: Extracellular superoxide dismutase
EPR: Electron paramagnetic resonance
Fe-MGD: *N*-methyl-d-glucaminedithiocarbamate (NO spin trap)
GC: Guanylate cyclase
VASP: Vasodilator-stimulated protein
WGA: Wheat germ agglutinin

## References

[1] Adachi T, Marklund SL (1989). Interactions between human extracellular superoxide dismutase C and sulfated polysaccharides. J Biol Chem.

[2] Armstrong SC (2004). Protein kinase activation and myocardial ischemia/reperfusion injury. Cardiovasc Res.

[3] Barouch LA, Harrison RW, Skaf MW, Rosas GO, Cappola TP, Kobeissi ZA, Hobai IA, Lemmon CA, Burnett AL, O’Rourke B, Rodriguez ER, Huang PL, Lima JA, Berkowitz DE, Hare JM (2002). Nitric oxide regulates the heart by spatial confinement of nitric oxide synthase isoforms. Nature.

[4] Bellamy TC, Wood J, Goodwin DA, Garthwaite J (2000). Rapid desensitization of the nitric oxide receptor, soluble guanylyl cyclase, underlies diversity of cellular cGMP responses. Proc Natl Acad Sci USA.

[5] Bolli R (2000). The late phase of preconditioning. Circ Res.

[6] Chen Y, Hou M, Li Y, Traverse JH, Zhang P, Salvemini D, Fukai T, Bache RJ (2005). Increased superoxide production causes coronary endothelial dysfunction and depressed oxygen consumption in the failing heart. Am J Physiol Heart Circ Physiol.

[7] Clarke R, Armitage J (2002). Antioxidant vitamins and risk of cardiovascular disease: review of large-scale randomised trials. Cardiovasc Drugs Ther.

[8] Csont T, Gorbe A, Bereczki E, Szunyog A, Aypar E, Toth ME, Varga ZV, Csonka C, Fulop F, Santha M, Ferdinandy P (2010). Biglycan protects cardiomyocytes against hypoxia/reoxygenation injury: role of nitric oxide. J Mol Cell Cardiol.

[9] Dai S, Yuan F, Mu J, Li C, Chen N, Guo S, Kingery J, Prabhu SD, Bolli R, Rokosh G (2010). Chronic AMD3100 antagonism of SDF-1alpha-CXCR4 exacerbates cardiac dysfunction and remodeling after myocardial infarction. J Mol Cell Cardiol.

[10] Di Lisa F, Bernardi P (2006). Mitochondria and ischemia-reperfusion injury of the heart: fixing a hole. Cardiovasc Res.

[11] Foster MW, McMahon TJ, Stamler JS (2003). *S*-nitrosylation in health and disease. Trends Mol Med.

[12] Fujio Y, Nguyen T, Wencker D, Kitsis RN, Walsh K (2000). Akt promotes survival of cardiomyocytes in vitro and protects against ischemia-reperfusion injury in mouse heart. Circulation.

[13] Fukai T, Siegfried MR, Ushio-Fukai M, Cheng Y, Kojda G, Harrison DG (2000). Regulation of the vascular extracellular superoxide dismutase by nitric oxide and exercise training. J Clin Invest.

[14] Fukai T, Siegfried MR, Ushio-Fukai M, Griendling KK, Harrison DG (1999). Modulation of extracellular superoxide dismutase expression by angiotensin II and hypertension. Circ Res.

[15] Goldstein S, Czapski G (1995). The reaction of NO· with O_2_^·-^ and HO_2_^·^: a pulse radiolysis study. Free Radic Biol Med.

[16] Gonzales GF, Chung FA, Miranda S, Valdez LB, Zaobornyj T, Bustamante J, Boveris A (2005). Heart mitochondrial nitric oxide synthase is upregulated in male rats exposed to high altitude (4,340 m). Am J Physiol Heart Circ Physiol.

[17] Gryglewski RJ, Palmer RM, Moncada S (1986). Superoxide anion is involved in the breakdown of endothelium-derived vascular relaxing factor. Nature.

[18] Gulick J, Subramaniam A, Neumann J, Robbins J (1991). Isolation and characterization of the mouse cardiac myosin heavy chain genes. J Biol Chem.

[19] Guo Y, Sanganalmath SK, Wu W, Zhu X, Huang Y, Tan W, Ildstad ST, Li Q, Bolli R (2012). Identification of inducible nitric oxide synthase in peripheral blood cells as a mediator of myocardial ischemia/reperfusion injury. Basic Res Cardiol.

[20] Guo Y, Wu WJ, Qiu Y, Tang XL, Yang Z, Bolli R (1998). Demonstration of an early and a late phase of ischemic preconditioning in mice. Am J Physiol.

[21] Hare JM (2003). Nitric oxide and excitation-contraction coupling. J Mol Cell Cardiol.

[22] Hausenloy DJ, Baxter G, Bell R, Botker HE, Davidson SM, Downey J, Heusch G, Kitakaze M, Lecour S, Mentzer R, Mocanu MM, Ovize M, Schulz R, Shannon R, Walker M, Walkinshaw G, Yellon DM (2010). Translating novel strategies for cardioprotection: the Hatter Workshop Recommendations. Basic Res Cardiol.

[23] Heger J, Godecke A, Flogel U, Merx MW, Molojavyi A, Kuhn-Velten WN, Schrader J (2002). Cardiac-specific overexpression of inducible nitric oxide synthase does not result in severe cardiac dysfunction. Circ Res.

[24] Heusch G, Boengler K, Schulz R (2008). Cardioprotection: nitric oxide, protein kinases, and mitochondria. Circulation.

[25] Hu X, Dai S, Wu W-J, Tan W, Zhu X, Mu J, Guo Y, Bolli R, Rokosh G (2007). Stromal cell derived factor-1 alpha confers protection against myocardial ischemia/reperfusion injury: role of the cardiac stromal cell derived factor-1 alpha CXCR4 axis. Circulation.

[26] Jolly SR, Kane WJ, Bailie MB, Abrams GD, Lucchesi BR (1984). Canine myocardial reperfusion injury: its reduction by the combined administration of superoxide dismutase and catalase. Circ Res.

[27] Karlsson K, Sandstrom J, Edlund A, Edlund T, Marklund SL (1993). Pharmacokinetics of extracellular-superoxide dismutase in the vascular system. Free Radic Biol Med.

[28] Keith WG, Powell RE (1969). Kinetics of decomposition of peroxynitrous acid. J Chem Soc A.

[29] Kliment CR, Suliman HB, Tobolewski JM, Reynolds CM, Day BJ, Zhu X, McTiernan CF, McGaffin KR, Piantadosi CA, Oury TD (2009). Extracellular superoxide dismutase regulates cardiac function and fibrosis. J Mol Cell Cardiol.

[30] Koppenol WH, Moreno JJ, Pryor WA, Ischiropoulos H, Beckman JS (1992). Peroxynitrite, a cloaked oxidant formed by nitric oxide and superoxide. Chem Res Toxicol.

[31] Li Q, Bolli R, Qiu Y, Tang XL, Guo Y, French BA (2001). Gene therapy with extracellular superoxide dismutase protects conscious rabbits against myocardial infarction. Circulation.

[32] Li Q, Bolli R, Qiu Y, Tang XL, Murphree SS, French BA (1998). Gene therapy with extracellular superoxide dismutase attenuates myocardial stunning in conscious rabbits. Circulation.

[33] Li Q, Guo Y, Wu WJ, Ou Q, Zhu X, Tan W, Yuan F, Chen N, Dawn B, Luo L, O’Brien E, Bolli R (2011). Gene transfer as a strategy to achieve permanent cardioprotection I: rAAV-mediated gene therapy with inducible nitric oxide synthase limits infarct size 1 year later without adverse functional consequences. Basic Res Cardiol.

[34] Lima B, Lam GK, Xie L, Diesen DL, Villamizar N, Nienaber J, Messina E, Bowles D, Kontos CD, Hare JM, Stamler JS, Rockman HA (2009). Endogenous *S*-nitrosothiols protect against myocardial injury. Proc Natl Acad Sci USA.

[35] Marklund SL (2002). Extracellular superoxide dismutase. Methods Enzymol.

[36] Marklund SL (1984). Extracellular superoxide dismutase and other superoxide dismutase isoenzymes in tissues from nine mammalian species. Biochem J.

[37] Marklund SL (1984). Extracellular superoxide dismutase in human tissues and human cell lines. J Clin Invest.

[38] Marklund SL (1992). Regulation by cytokines of extracellular superoxide dismutase and other superoxide dismutase isoenzymes in fibroblasts. J Biol Chem.

[39] Mery PF, Pavoine C, Belhassen L, Pecker F, Fischmeister R (1993). Nitric oxide regulates cardiac Ca^2+^ current: involvement of cGMP-inhibited and cGMP-stimulated phosphodiesterases through guanylyl cyclase activation. J Biol Chem.

[40] Michaelides AP, Andrikopoulos GK, Oikonomou EV, Psomadaki ZD, Richter DJ, Dilaveris PE, Exadaktylos NI, Stefanadis CI, Toutouzas PK (2003). Improved myocardial performance during repetitive exercise testing: the role of extracellular superoxide dismutase activity in a model of exercise-induced myocardial preconditioning. Am Heart J.

[41] Ookawara T, Kizaki T, Takayama E, Imazeki N, Matsubara O, Ikeda Y, Suzuki K, Li Ji L, Tadakuma T, Taniguchi N, Ohno H (2002). Nuclear translocation of extracellular superoxide dismutase. Biochem Biophys Res Commun.

[42] Oury TD, Card JP, Klann E (1999). Localization of extracellular superoxide dismutase in adult mouse brain. Brain Res.

[43] Oury TD, Day BJ, Crapo JD (1996). Extracellular superoxide dismutase: a regulator of nitric oxide bioavailability. Lab Invest.

[44] Peng T, Yang D (2010). HKGreen-3: a rhodol-based fluorescent probe for peroxynitrite. Org Lett.

[45] Qi X, Pohl NM, Loesch M, Hou S, Li R, Qin JZ, Cuenda A, Chen G (2007). p38alpha antagonizes p38gamma activity through c-Jun-dependent ubiquitin-proteasome pathways in regulating Ras transformation and stress response. J Biol Chem.

[46] Rubanyi GM, Vanhoutte PM (1986). Oxygen-derived free radicals, endothelium, and responsiveness of vascular smooth muscle. Am J Physiol.

[47] Sandstrom J, Karlsson K, Edlund T, Marklund SL (1993). Heparin-affinity patterns and composition of extracellular superoxide dismutase in human plasma and tissues. Biochem J.

[48] Schulz R, Belosjorow S, Gres P, Jansen J, Michel MC, Heusch G (2002). p38 MAP kinase is a mediator of ischemic preconditioning in pigs. Cardiovasc Res.

[49] Schulz R, Gres P, Skyschally A, Duschin A, Belosjorow S, Konietzka I, Heusch G (2003). Ischemic preconditioning preserves connexin 43 phosphorylation during sustained ischemia in pig hearts in vivo. FASEB J.

[50] Schulz R, Kelm M, Heusch G (2004). Nitric oxide in myocardial ischemia/reperfusion injury. Cardiovasc Res.

[51] Schulz R, Rassaf T, Massion PB, Kelm M, Balligand JL (2005). Recent advances in the understanding of the role of nitric oxide in cardiovascular homeostasis. Pharmacol Ther.

[52] Schwartz Longacre L, Kloner RA, Arai AE, Baines CP, Bolli R, Braunwald E, Downey J, Gibbons RJ, Gottlieb RA, Heusch G, Jennings RB, Lefer DJ, Mentzer RM, Murphy E, Ovize M, Ping P, Przyklenk K, Sack MN, Vander Heide RS, Vinten-Johansen J, Yellon DM, Blood Institute NIoH (2011). New horizons in cardioprotection: recommendations from the 2010 National Heart, Lung, and Blood Institute Workshop. Circulation.

[53] Shinobu LA, Jones SG, Jones MM (1984). Sodium *N*-methyl-d-glucamine dithiocarbamate and cadmium intoxication. Acta Pharmacol Toxicol (Copenh).

[54] Sjoquist PO, Carlsson L, Jonason G, Marklund SL, Abrahamsson T (1991). Cardioprotective effects of recombinant human extracellular-superoxide dismutase type C in rat isolated heart subjected to ischemia and reperfusion. J Cardiovasc Pharmacol.

[55] Smolenski A, Bachmann C, Reinhard K, Honig-Liedl P, Jarchau T, Hoschuetzky H, Walter U (1998). Analysis and regulation of vasodilator-stimulated phosphoprotein serine 239 phosphorylation in vitro and in intact cells using a phosphospecific monoclonal antibody. J Biol Chem.

[56] Smolenski A, Poller W, Walter U, Lohmann SM (2000). Regulation of human endothelial cell focal adhesion sites and migration by cGMP-dependent protein kinase I. J Biol Chem.

[57] Stralin P, Marklund SL (2001). Vasoactive factors and growth factors alter vascular smooth muscle cell EC-SOD expression. Am J Physiol Heart Circ Physiol.

[58] Szelid Z, Pokreisz P, Liu X, Vermeersch P, Marsboom G, Gillijns H, Pellens M, Verbeken E, Van de Werf F, Collen D, Janssens SP (2010). Cardioselective nitric oxide synthase 3 gene transfer protects against myocardial reperfusion injury. Basic Res Cardiol.

[59] van Deel ED, Lu Z, Xu X, Zhu G, Hu X, Oury TD, Bache RJ, Duncker DJ, Chen Y (2008). Extracellular superoxide dismutase protects the heart against oxidative stress and hypertrophy after myocardial infarction. Free Radic Biol Med.

[60] Wahlund G, Marklund SL, Sjoquist PO (1992). Extracellular-superoxide dismutase type C (EC-SOD C) reduces myocardial damage in rats subjected to coronary occlusion and 24 hours of reperfusion. Free Radic Res Commun.

[61] Wang P, Zweier JL (1996). Measurement of nitric oxide and peroxynitrite generation in the postischemic heart: evidence for peroxynitrite-mediated reperfusion injury. J Biol Chem.

[62] West MB, Rokosh G, Obal D, Velayutham M, Xuan YT, Hill BG, Keith RJ, Schrader J, Guo Y, Conklin DJ, Prabhu SD, Zweier JL, Bolli R, Bhatnagar A (2008). Cardiac myocyte-specific expression of inducible nitric oxide synthase protects against ischemia/reperfusion injury by preventing mitochondrial permeability transition. Circulation.

[63] Whalen EJ, Foster MW, Matsumoto A, Ozawa K, Violin JD, Que LG, Nelson CD, Benhar M, Keys JR, Rockman HA, Koch WJ, Daaka Y, Lefkowitz RJ, Stamler JS (2007). Regulation of beta-adrenergic receptor signaling by *S*-nitrosylation of G-protein-coupled receptor kinase 2. Cell.

[64] Xu L, Eu JP, Meissner G, Stamler JS (1998). Activation of the cardiac calcium release channel (ryanodine receptor) by poly-*S*-nitrosylation. Science.

[65] Yamashita T, Kawashima S, Ohashi Y, Ozaki M, Rikitake Y, Inoue N, Hirata K, Akita H, Yokoyama M (2000). Mechanisms of reduced nitric oxide/cGMP-mediated vasorelaxation in transgenic mice overexpressing endothelial nitric oxide synthase. Hypertension.

[66] Zanella B, Giordano E, Muscari C, Zini M, Guarnieri C (2004). Nitric oxide synthase activity in rat cardiac mitochondria. Basic Res Cardiol.

[67] Zweier JL, Wang P, Kuppusamy P (1995). Direct measurement of nitric oxide generation in the ischemic heart using electron paramagnetic resonance spectroscopy. J Biol Chem.

