# Supplementary material for: Cardiomyocyte-restricted overexpression of extracellular superoxide dismutase increases nitric oxide bioavailability and reduces infarct size after ischemia/reperfusion
Source: Basic Res Cardiol. 2012 Oct 26;107(6):305. doi: 10.1007/s00395-012-0305-1 (PMC3505528; doi:10.1007/s00395-012-0305-1)
Supplement: Supplementary file 1 — Supplement Figure 1. EcSOD protein expression in isolated cardiomyocytes and whole heart from WT and ecSOD Tg mice detected by Western analysis (panel A); Representative Western-blots and quantitative analysis of eNOS expression in whole heart from WT and ecSOD Tg mice detected by Western analysis (n = 3, panel B). iNOS expression in whole heart from WT and ecSOD Tg mice. Protein samples from fresh isolated lung and heart tissue of cardiomyocyte-specific iNOS overexpressing mice [23] served as positive control. GAPDH was used as loading control (panel C)Supplement Figure 2. Confocal microscopy images of isolated cardiomyocytes of WT (A) and ecSOD Tg mice (B) after 30 min of hypoxia and 15 min of reoxygenation. Experiments were performed after 1 h incubation in HKGreen-3 (5 μM), Mitosox Red (5 μM) and Hoechst stain (5 μM)Supplement Figure 3. ERK and AKT signaling in isolated Langendorff-mode perfused WT and ecSOD Tg hearts. Phospho- and total levels of AKT (panel A) ERK (panel B) were measured in hearts of WT and ecSOD Tg mice after 30 min global ischemia and 90 min reperfusion. Representative Western blots for summary data (panel C). Total kinase levels were normalized to GAPDH and phospho-kinase levels were normalized to total kinase. Values are the mean ± SEM (n = 3). *P < 0.05 vs. WT [file 395_2012_305_MOESM1_ESM.pptx]

## Slide 1
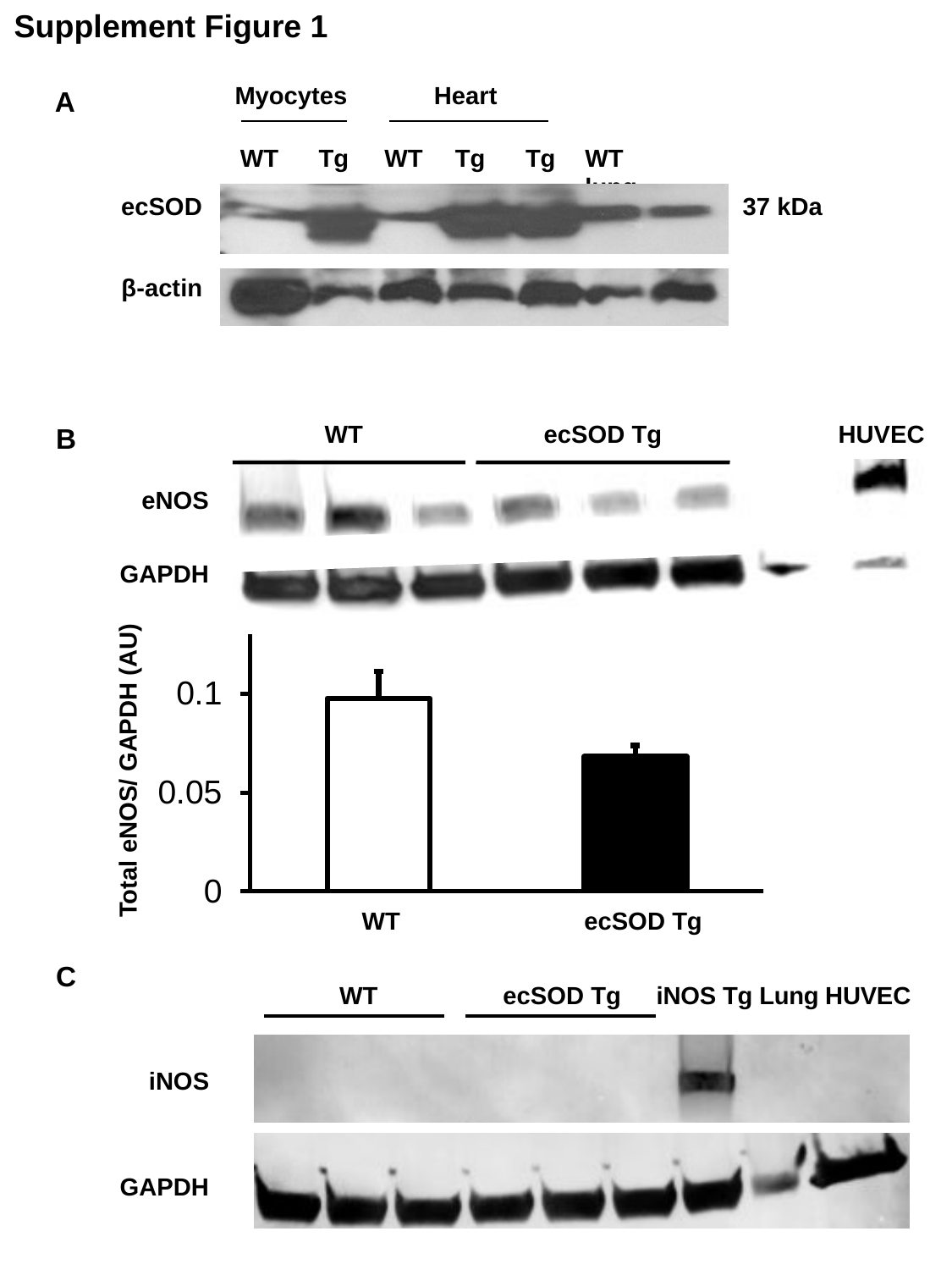

Supplement Figure 1
Myocytes
Heart
A
 WT
Tg
Tg
Tg
WT lung
WT
ecSOD
37 kDa
β-actin
WT
ecSOD Tg
HUVEC
B
eNOS
GAPDH
Total eNOS/ GAPDH (AU)
WT
ecSOD Tg
C
 WT ecSOD Tg iNOS Tg Lung HUVEC
iNOS
GAPDH

## Slide 2
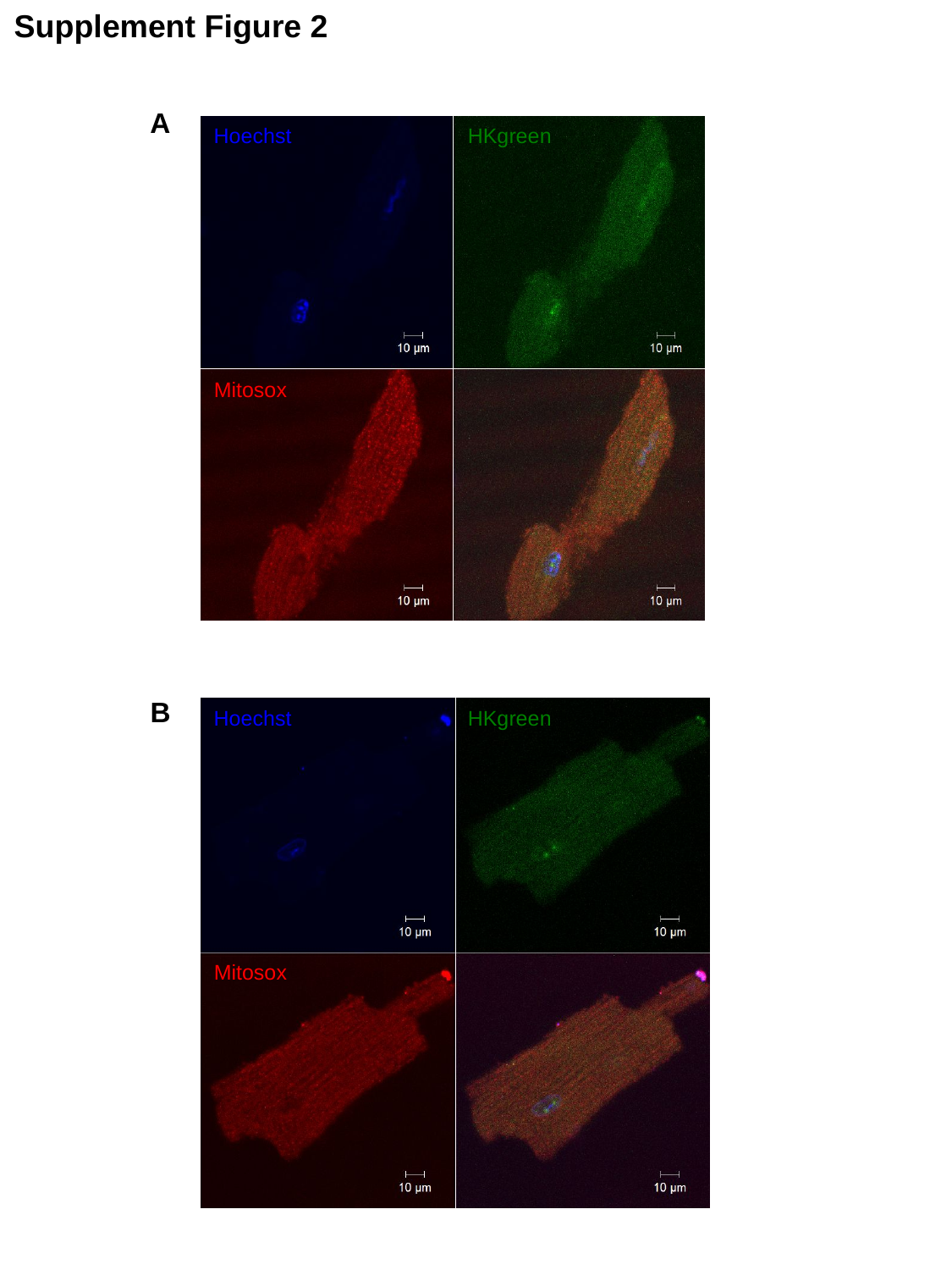

Supplement Figure 2
A
Hoechst
HKgreen
Mitosox
B
Hoechst
HKgreen
Mitosox

## Slide 3
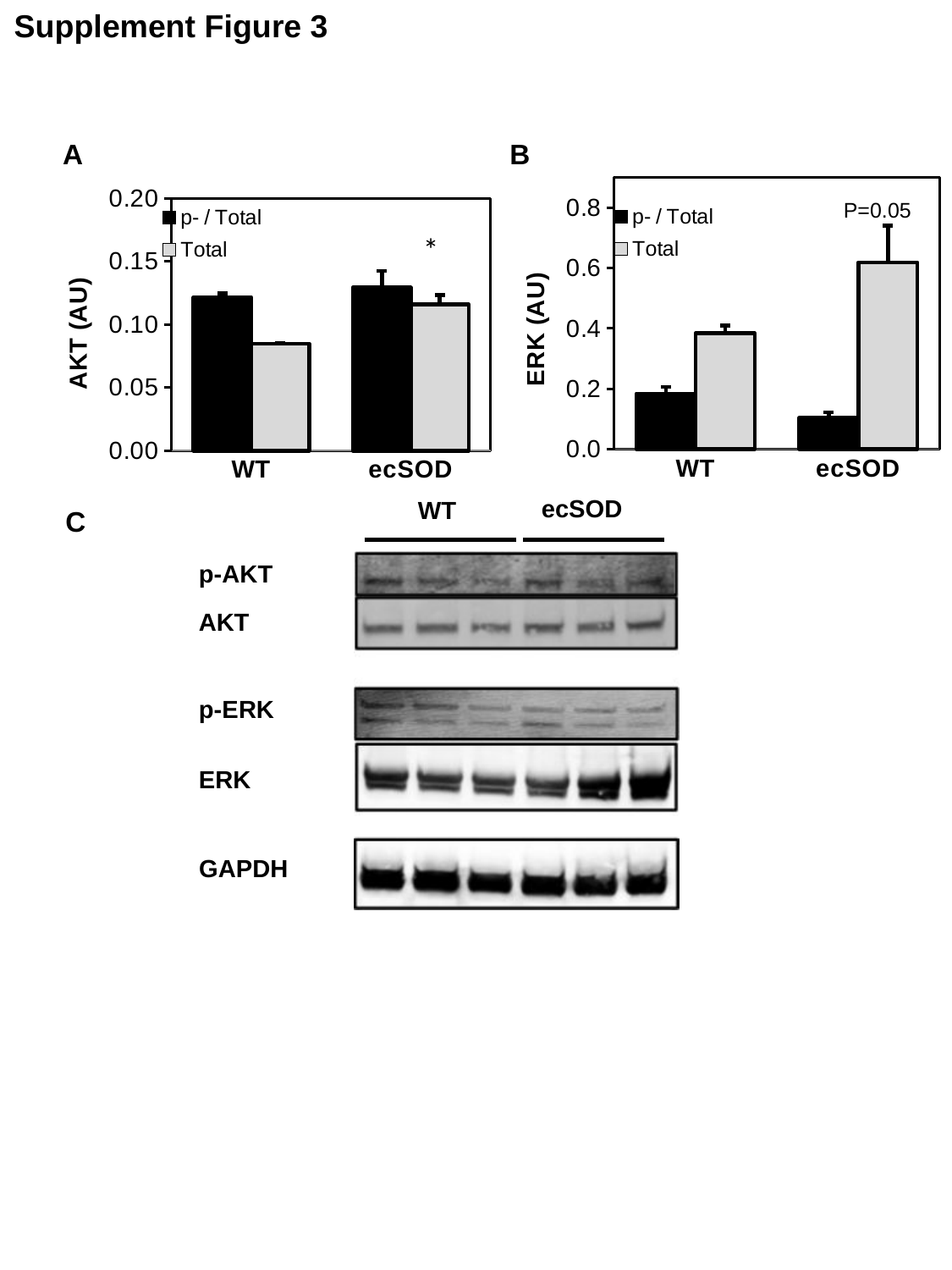

Supplement Figure 3
A
B
### Chart
| Category | | |
|---|---|---|
| WT | 0.1840831768732737 | 0.3837600341805063 |
| ecSOD | 0.10312860687402386 | 0.6184367065446605 |
### Chart
| Category | | |
|---|---|---|
| WT | 0.1215364225912382 | 0.08475990045158555 |
| ecSOD | 0.1291863651504779 | 0.11575253229493675 |P=0.05
*
ecSOD
WT
C
p-AKT
AKT
p-ERK
ERK
GAPDH
